# Evidence for long-term sensitization of the bowel in patients with post-infectious-IBS

**DOI:** 10.1038/s41598-017-12618-7

**Published:** 2017-10-19

**Authors:** D. Balemans, S. U. Mondelaers, V. Cibert-Goton, N. Stakenborg, J. Aguilera-Lizarraga, J. Dooley, A. Liston, D. C. Bulmer, P. Vanden Berghe, G. E. Boeckxstaens, M. M. Wouters

**Affiliations:** 1 Translational Research Center for Gastrointestinal Disorders, Dept. of Clinical and Experimental Medicine, KU Leuven, Leuven, Belgium; 2 National Centre for Bowel Research and Surgical Innovation, Centre for Neuroscience and Trauma, Blizard Institute, Bart’s and the London school of Medicine and Dentistry, Queen Mary University of London, London, UK; 3 Autoimmune Genetics Laboratory, VIB and Department of Microbiology and Immunology, KU Leuven, Belgium; 4 Department of Pharmacology, University of Cambridge, Cambridge, UK

## Abstract

Post-infectious irritable bowel syndrome (PI-IBS) is a common gastrointestinal disorder characterized by persistent abdominal pain despite recovery from acute gastroenteritis. The underlying mechanisms are unclear, although long-term changes in neuronal function, and low grade inflammation of the bowel have been hypothesized. We investigated the presence and mechanism of neuronal sensitization in a unique cohort of individuals who developed PI-IBS following exposure to contaminated drinking water 7 years ago. We provide direct evidence of ongoing sensitization of neuronal signaling in the bowel of patients with PI-IBS. These changes occur in the absence of any detectable tissue inflammation, and instead appear to be driven by pro-nociceptive changes in the gut micro-environment. This is evidenced by the activation of murine colonic afferents, and sensitization responses to capsaicin in dorsal root ganglia (DRGs) following application of supernatants generated from tissue biopsy of patients with PI-IBS. We demonstrate that neuronal signaling within the bowel of PI-IBS patients is sensitized 2 years after the initial infection has resolved. This sensitization appears to be mediated by a persistent pro-nociceptive change in the gut micro-environment, that has the capacity to stimulate visceral afferents and facilitate neuronal TRPV1 signaling.

## Introduction

Infectious gastroenteritis (IGE) is a significant risk factor in the development of irritable bowel syndrome (IBS), a chronic functional gastrointestinal disorder, characterized by abdominal pain and altered bowel habit in the absence of ongoing organic pathology. Up to 36% of patients with gastroenteritis may go on to develop post-infectious IBS (PI-IBS). The majority of PI-IBS patients suffer from increased visceral pain perception or visceral hypersensitivity (VHS) suggesting that long term changes in pain processing occur in PI-IBS^1^. The underlying mechanisms are still not clear, however it has been proposed that an incomplete resolution of the immune response leads to a persistent microscopic inflammation of the bowel, facilitating activation and sensitization of pain sensing nerves^2,3^.

Gwee *et al*. indeed demonstrated increased IL-1β mRNA expression in rectal biopsies following an episode of acute IGE^4^, while enhanced cytokine release (TNF-α, IL-1β and IL-6) by circulating PI-IBS lymphocytes is reported^5^. In addition, enterochromaffin cell numbers, intraepithelial lymphocytes and intestinal permeability are increased up to one year after *Campylobacter* enteritis^6^. Moreover, mast cell numbers are increased in the terminal ileum of PI-IBS patients following *Shigella* gastroenteritis^7^. Mediators released from these cells such as histamine, IL-1β, IL-6 and TNF-α stimulate or sensitize visceral nociceptors consistent with the hypothesis that nociceptor activation by low grade inflammation underpins abdominal pain in PI-IBS^8–10^. Consequently, there is great interest in identifying which specific ion channels are responsible for transducing the depolarization of nociceptors in response to inflammatory mediators.

Despite this compelling evidence, direct functional data from human studies of sensitized neuroimmune signaling in PI-IBS is lacking. The recent failure of 2 large-scale clinical trials with the anti-inflammatory drug mesalazine to show symptom improvement in diarrhea predominant IBS (IBS-D) highlights the urgent need to substantiate this hypothesis. We have recently developed the technical capacity to image calcium transients in human enteric neurons^11,12^ obtained from gut biopsies, and test the effect of supernatants generated from these biopsies on sensory nerve activity^12^. We are therefore in the position to examine both, changes in the function of a patient’s own neurons, and changes in the microenvironment of the patient’s bowel, thereby putting our group in the unique position to confirm the sensitization of neuronal signaling in PI-IBS patients. To do this, we have obtained samples from PI-IBS patients, who were exposed to contaminated tap water, 2 years previously^13^, allowing us to study a unique cohort of PI-IBS patients for whom the duration of disease, and precipitating infectious event, are identical, greatly reducing intra-subject variability.

The aim of this study was to examine neuronal sensitivity in patients with PI-IBS, and to investigate the role of low-grade inflammation in these changes. To our surprise, we found no evidence for low grade inflammation in the bowel of these patients. However, we did find clear evidence for both neuronal sensitization in PI-IBS patients, and a shift in the bowel microenvironment to a pro-nociceptive state. Taken together, our data show for the first-time direct evidence of aberrant neuronal signaling in PI-IBS. Notably, this sensitization of gut function, is not mediated by persistent low grade inflammation, but instead is appears to be mediated by other pro-nociceptive changes in the mucosal micro-environment which modulate TRPV1 signaling.

## Results

### Sensitization of TRPV1 on submucosal neurons in PI-IBS

**Figure 1:**
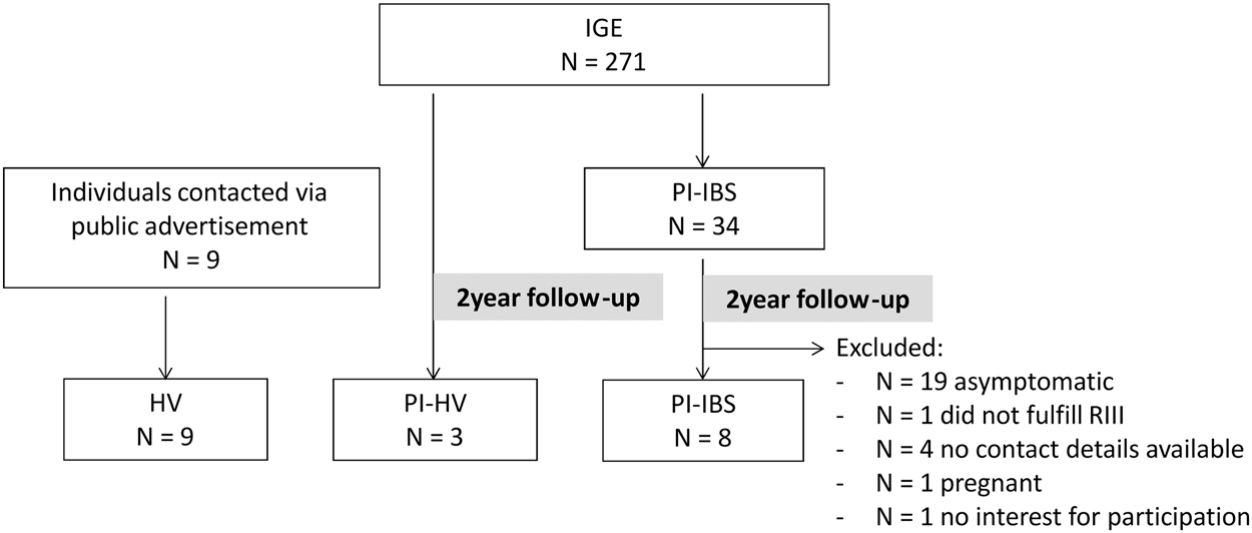
Flowchart of PI-IBS cohort. HV = healthy volunteer, IGE = infectious gastroenteritis, PI-HV = post-infectious healthy volunteer, PI-IBS = post-infectious IBS.

To investigate the underlying mechanism of PI-IBS, 8 patients were recruited 2 years following an outbreak in 2 Belgian villages^13^ (Fig. 1). In addition, 9 healthy volunteers (HVs) were recruited by public advertisement. Of note, 3 individuals who suffered from an infection but did not develop PI-IBS were also recruited (PI-HVs). Demographic data are summarized in Table 1.

**Table 1:** Demographic information on PI-IBS, PI-HV and HV subjects.

	HV	PI-HV	PI-IBS	p value (PI-IBS vs PI-HV)	p value (HV vs. PI-IBS)
N	9	3	8		
Male/Female	5/4 (44.4% F)	3/0 (0% F)	1/7 (87.5% F)	0.02	0.29
Age (median years[IQR])	45 [25–50]	61 [28–64]	53 [35–54]	0.37	0.21
IBS-D, n (%)	/	/	2 (25%)		
IBS-C, n (%)	/	/	0 (0%)		
IBS-A, n (%)	/	/	2 (25%)		
IBS-U, n (%)	/	/	4 (50%)		

F = female, HV = healthy volunteer, PI-IBS = post-infectious irritable bowel syndrome, PI-HV = post-infectious healthy volunteer, IBS-A = alternating type IBS, IBS-C = constipation predominant IBS, IBS-D = diarrhea predominant IBS, IBS-U = unclassified IBS, IQR = interquartile range. Statistics: Unpaired t-test (Age), Fisher’s exact test (Gender).

**Figure 2:**
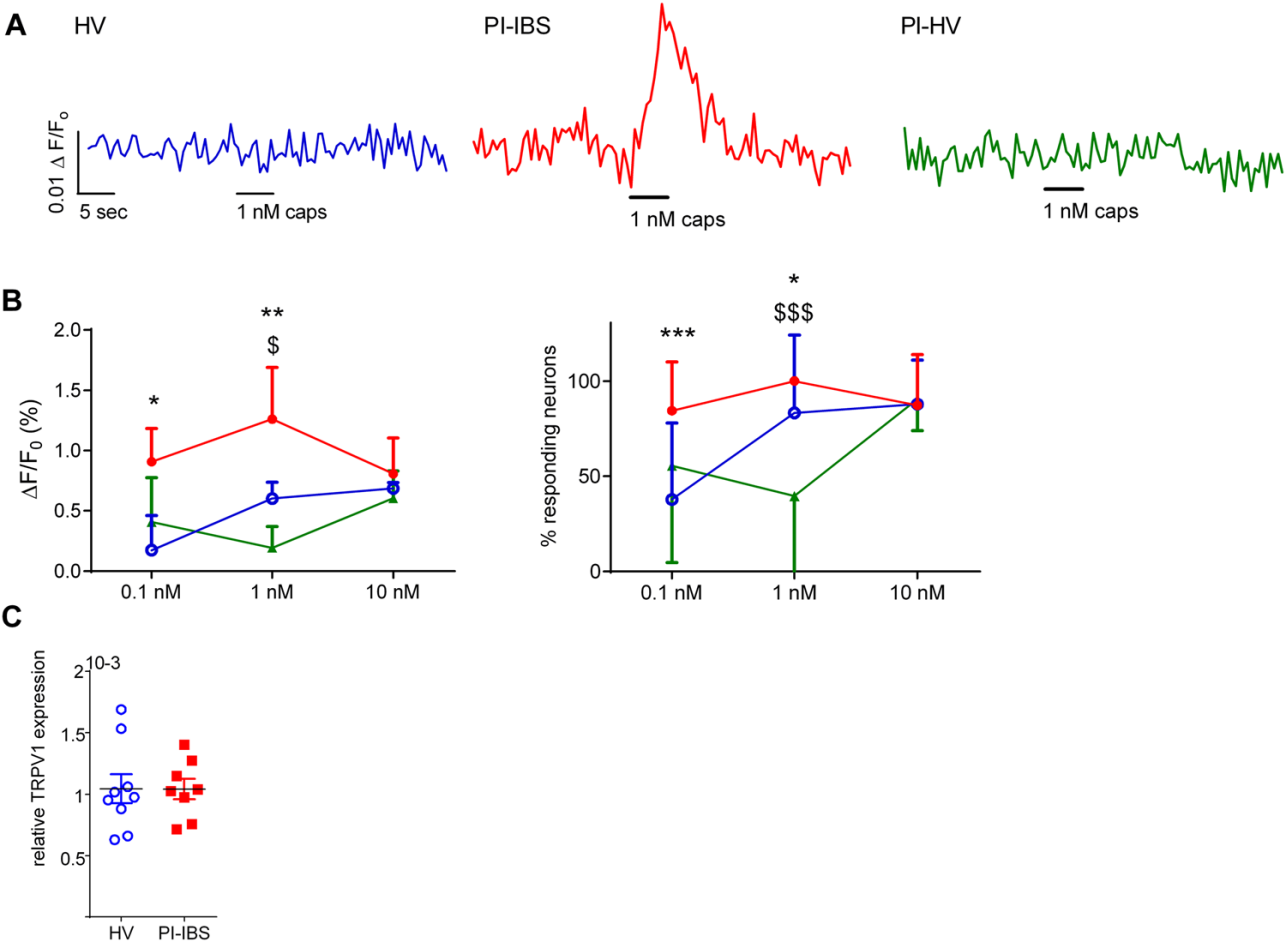
TRPV1 on human submucosal neurons of PI-IBS patients is more sensitive compared to healthy volunteers and post-infectious healthy volunteers. (**A**) Representative traces of the intracellular Ca^2+^ response of human submucosal neurons in biopsies of healthy volunteers (HV, blue) (n = 7), IBS patients (red) (n = 6) and PI-HV (green) (n = 3) to acute application of capsaicin (1 nM). (**B**) Data showing the amplitude of the Ca^2+^ flux (%) and the number of responding neurons to capsaicin (%) in PI-IBS patients (red, n = 6), HV (blue, n = 7) and PI-HV (green, n = 3). Data are presented as median + interquartile range (left) and mean + SD (right). *p < 0.05, **p < 0.01, ***p < 0.001 (PI-IBS vs. HV); ^$^p < 0.05, ^$$$^p < 0.001 (PI-IBS vs PI-HV), Two-way ANOVA (left) and Fisher’s exact test (right). (**C**) Relative mRNA expression for TRPV1 normalized to β-actin in rectal biopsies of HV (n = 9) and PI-IBS (n = 8) patients. Statistical analysis by Mann-Whitney U test. HV = healthy volunteer, PI-IBS = post-infectious irritable bowel syndrome, PI-HV = post-infectious healthy volunteer, TRP = transient receptor potential.

Inflammation enhances pain perception via the activation and/or sensitization of TRP channels, including TRPV1^14,15^, and we hypothesized that the persistence of a low-grade inflammation of the bowel in PI-IBS could lead to long term TRPV1 sensitization. Therefore, we performed live Ca^2+^ imaging of submucosal neurons in rectal biopsies from PI-IBS patients, HVs and PI-HVs^12^. Application of 0.1 and 1 nM capsaicin induced significantly greater Ca^2+^ fluxes in submucosal neurons from PI-IBS compared with HVs (0.1 nM: p = 0.012, 1 nM: p = 0.026) or PI-HVs (1 nM: p = 0.024) (Fig. 2A,B). In addition, exposure to 0.1 and 1 nM capsaicin activated significantly more submucosal neurons in PI-IBS compared with HVs (0.1 nM: p < 0.001, 1 nM: p = 0.017) and PI-HVs (1 nM: p < 0.001) (Fig. 2A,B). Responses to capsaicin were blocked by pre-treatment with the selective TRPV1 antagonist SB-366791 (1 µM) confirming specificity for TRPV1 (Supplementary Figure 1).

To assess if the increased Ca^2+^ response to capsaicin was mediated by increased expression of TRPV1 in PI-IBS, TRPV1 transcript levels in HVs and PI-IBS biopsies were compared. No significant difference was seen between TRPV1 mRNA expression in PI-IBS versus HVs biopsies (Fig. 2C), suggesting that the increased Ca^2+^ response to capsaicin was due to sensitization of TRPV1 channel function, rather than an increase in channel number.

### Biopsy supernatants from PI-IBS patients stimulate murine colonic afferents and DRGs

**Figure 3:**
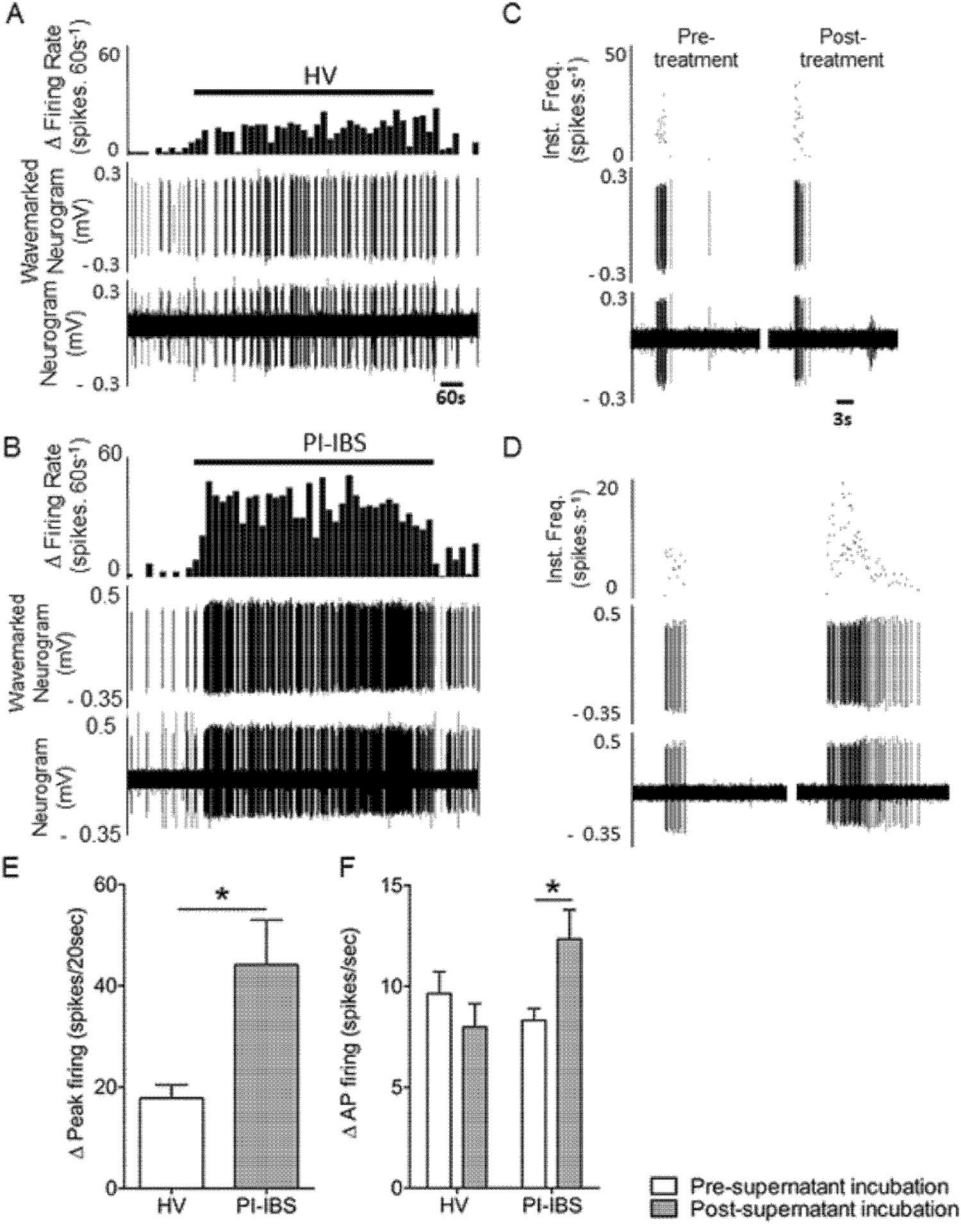
Rectal biopsy supernatants from PI-IBS patients directly activate and increase the mechanosensitivity of serosal lumbar splanchnic afferents. A and B are representative examples of supernatant-induced single serosal fiber response profiles over 12 minutes ring application (indicated as a horizontal bar), respectively from a HV and a PI-IBS biopsy. From top to bottom as successively depicted the firing rate (0.60 s^−1^), the wavemarked neurogram (mV), and the raw neurogram (mV). Averaged peak change in activity during HV (n = 8) or PI-IBS (n = 8) supernatant incubation are summarized as histograms in (**E**). Data are presented as mean ± SEM and significant difference indicated (Unpaired t-test, *p < 0.05). Mechanical probes with von Frey hair (600 mg) were performed prior to and after supernatant application. (**C** and **D**) are representative responses to single 3 seconds von Frey hair probing of the corresponding receptive field successively before (left panels) and after (right panels) supernatant incubation. Top panels are instantaneous frequency (Hz), middle and bottom panels are as previously described. Changes in mechanosensitivity (HV: n = 8 and PI-IBS: n = 8) are summarized as histograms in (**F**). Data are presented as mean ± SEM and significant difference indicated (Paired t-test, *p < 0.05). Hz = hertz, HV = healthy volunteer, PI-IBS = post-infectious irritable bowel syndrome.

Having shown that neuronal activity is sensitized in PI-IBS, we next sought to confirm the pro-nociceptive potential of the bowel in PI-IBS patients. Therefore, we examined the effect of tissue biopsy supernatants from PI-IBS patients and HVs on the activity of mouse colonic afferents thought to play a role in visceral nociception, and TRPV1 signaling in DRGs. Application of supernatants from PI-IBS biopsies to the mucosal surface below the receptive field of colonic afferents produced a robust increase in nerve discharge compared with supernatant from HV biopsies (Fig. 3A,B,E) (mean peak increase in firing PI-IBS (n = 8) 2.2 ± 0.4 Hz compared with HV (n = 8) 0.9 ± 0.1 Hz, p = 0.02). Furthermore, responses to von Frey hair (vfH) probing of the receptive field were significantly sensitized following removal of the PI-IBS supernatant, (8.3 ± 0.6 Hz pre-incubation vs. 12.3 ± 1.5 Hz post-incubation; p = 0.016, n = 8) (Fig. 3D,F), and unchanged following removal of supernatant from HV biopsies (9.6 ± 1.1 Hz pre-incubation vs. 8.0 ± 1.1 Hz post-incubation, n = 8, Fig. 3C,F). These findings demonstrating a clear pro-nociceptive change in the bowel from PI-IBS patients.

**Figure 4:**
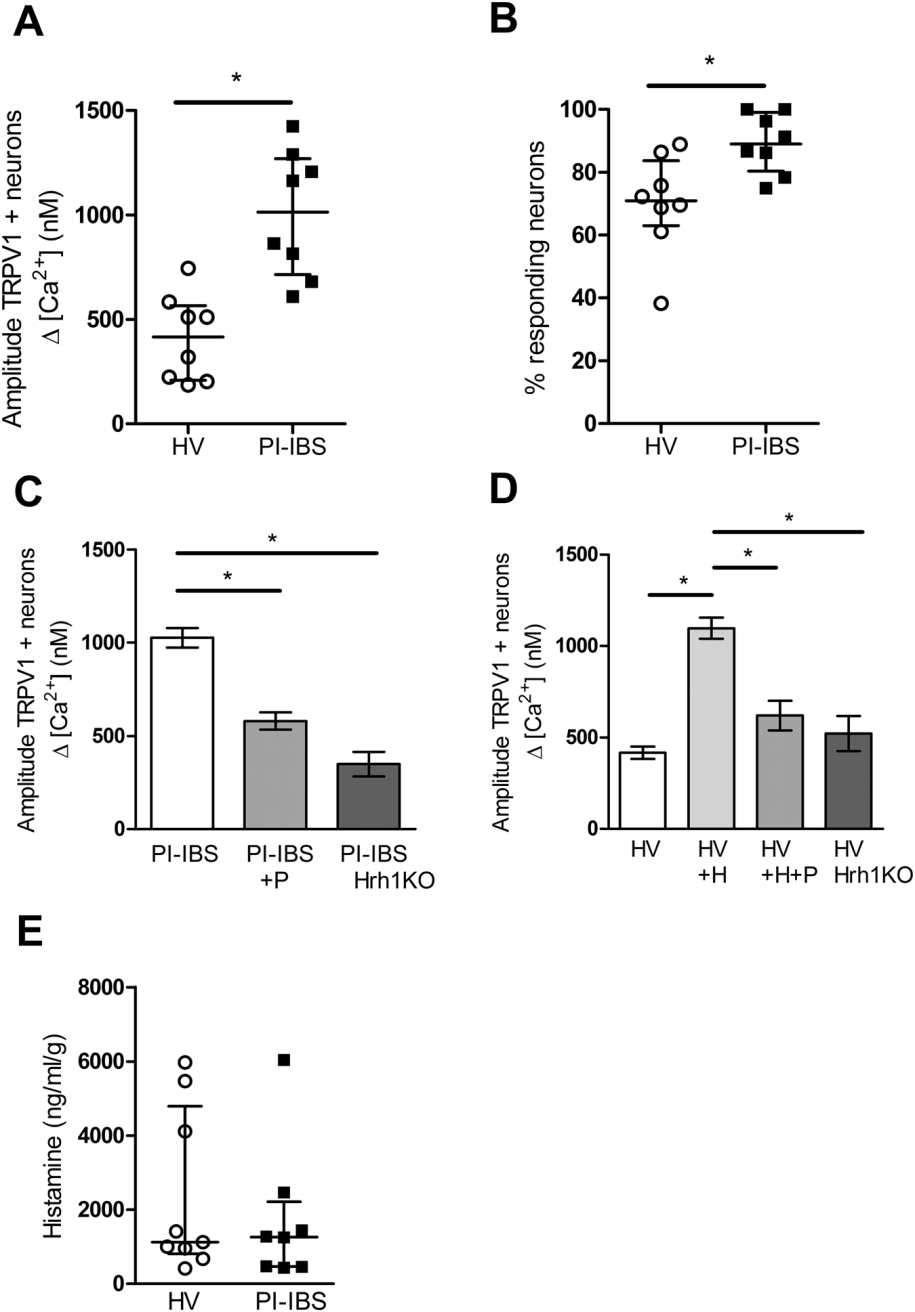
Rectal biopsy supernatants from PI-IBS patients sensitize TRPV1 on murine DRG neurons mediated by histamine via the Hrh1. Effect of overnight incubation of murine DRG neurons with supernatant of cultured rectal biopsies of PI-IBS patients (n = 8) or HV (n = 8) on the Ca^2+^ response (**A**) and percentage of responding neurons (**B**) to capsaicin (10 nM). Data are shown as median + interquartile range. (*p < 0.001, Mann Whitney U test). (**C**,**D**) Data showing the capsaicin-induced Ca^2+^ response after overnight incubation with supernatant of cultured rectal biopsies of PI-IBS patients (n = 8) in the presence or absence of pyrilamine (1 µM) (+P) (n = 8) and in histamine 1 receptor knock-out (Hrh1 KO) mice (n = 5) (**C**) Effect of overnight incubation with HV supernatants (n = 8) supplemented with histamine (10 µM) (+H) (n = 8) or histamine (10 µM) and pyrilamine (1 µM) (+H + P) (n = 4) or in Hrh1 KO mice (n = 5) on the Ca^2+^ response to capsaicin (**D**). Data are shown as mean + standard error of the mean (SEM). *p < 0.001 Statistical analysis by One-way ANOVA (**E**) Histamine levels were normalized to the weight of biopsy and compared in rectal biopsy supernatants of HV (n = 9) versus PI-IBS (n = 8) patients. Mann-Whitney U test. H = histamine, HV = healthy volunteer, P = pyrilamine, PI-IBS = post-infectious irritable bowel syndrome, Hrh1 KO = histamine 1 receptor knock-out.

Consistent with this change, DRG neurons incubated with PI-IBS supernatants demonstrated significantly greater Ca^2+^ responses to capsaicin compared to neurons incubated with HV supernatants (Fig. 4A), and significantly more DRG neurons responded to capsaicin following incubation with PI-IBS supernatants compared with HV supernatants (Fig. 4B). Taken together, this data demonstrates the presence of bioactive mediators in the bowel of patients with PI-IBS, which have the potential to directly activate colonic afferents and sensitize TRPV1 channels, potentially contributing to VHS in PI-IBS.

### Lack of evidence for persistent low grade inflammation or increased cytokine release in PI-IBS

**Figure 5:**
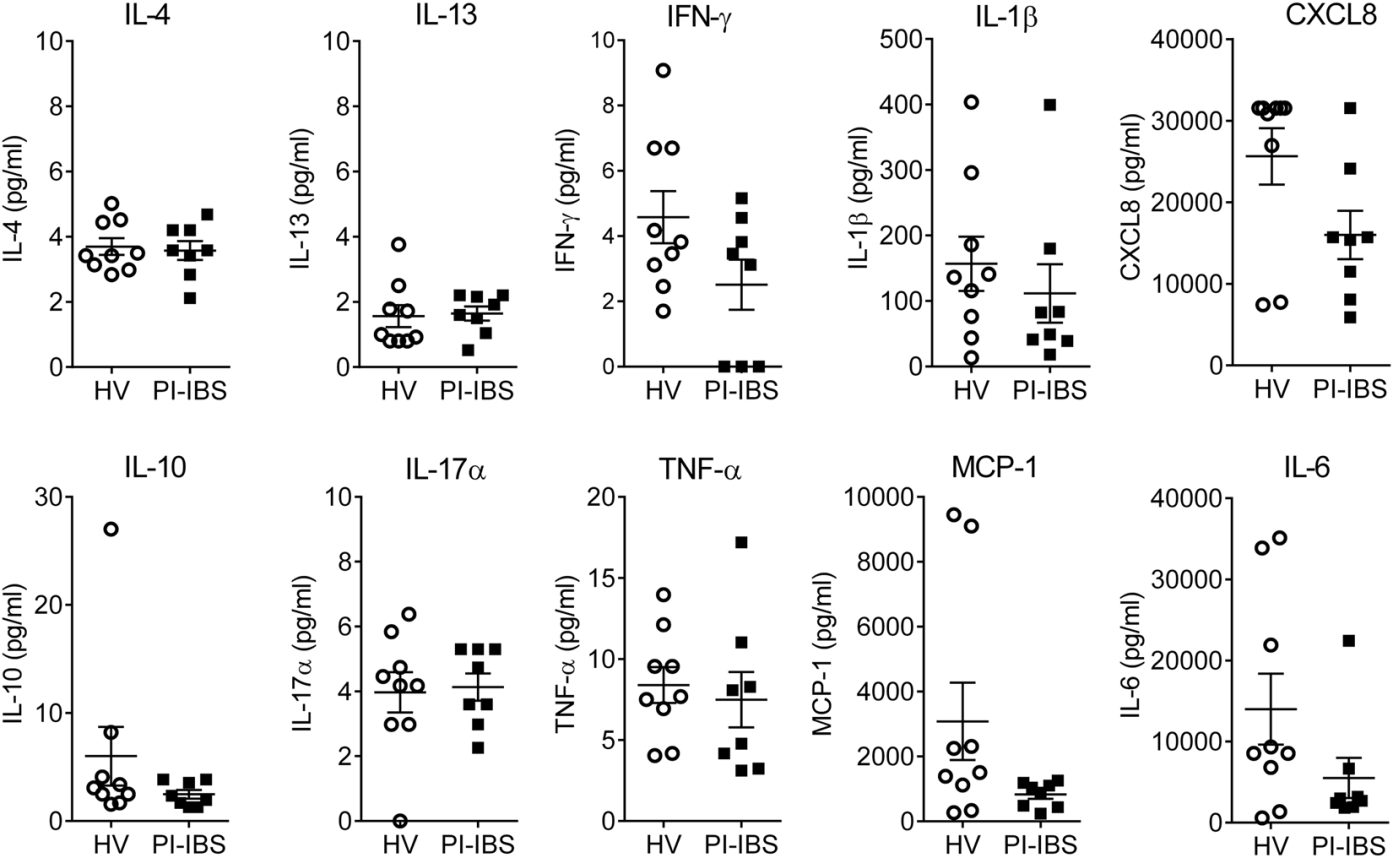
Cytokine levels in rectal biopsy supernatants are similar between HV and PI-IBS patients. The amount of inflammatory cytokines was evaluated in rectal biopsy supernatants of HV (n = 9) and PI-IBS (n = 8) patients. Statistical analysis by Mann-Whitney U test. HV = healthy volunteer, IFN = interferon, IL = interleukin, MCP-1 = monocyte chemoattractant protein-1, PI-IBS = post-infectious IBS, TNF = tumor necrosis factor.

**Figure 6:**
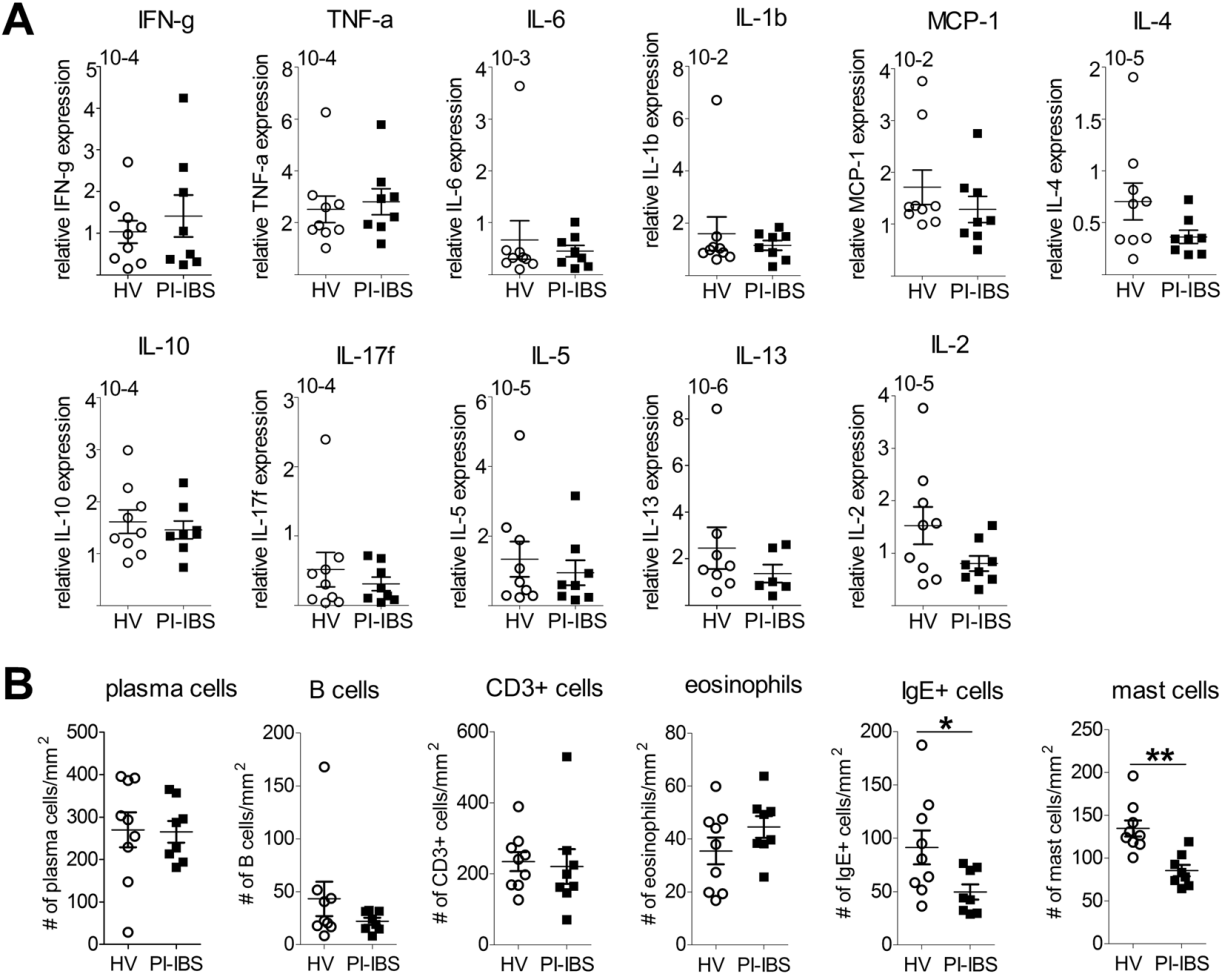
No evidence for low-grade inflammation in rectal biopsy samples of PI-IBS patients. (**A**) Relative mRNA expression for inflammatory markers normalized to β-actin in rectal biopsies of HV (n = 9) and PI-IBS (n = 8) patients. (**B**) Counting of plasma cells, B cells, CD3^+^ T cells, eosinophils, IgE^+^ cells and mast cells, in HV (n = 9) and PI-IBS (n = 8) rectal biopsies (as indicated). Mann-Whitney U test, *p < 0.05; **p < 0.01. HV = healthy volunteer, IFN = interferon, IL = interleukin, MCP-1 = monocyte chemoattractant protein-1, PI-IBS = post-infectious IBS, TNF = tumor-necrosis factor.

To understand the identity of these mediators we looked at the expression of pro-inflamamatory cytokines in biopsy samples as persistent low grade inflammation of the bowel has been proposed to underlie the production of pain in PI-IBS. Consistent with this hypothesis several cytokines e.g. IL-1β, IL-2 and IL-10 have been shown to stimulate colonic afferents^9,10^, and TNF-α sensitizes afferent endings and DRG neurons via TRPV1 or TRPA1 channels^10,16^. However, measurement of cytokine levels in biopsy supernatants failed to show any difference in *IL-4*, *IL-13*, *IFN-γ*, *IL-1β*, *CXCL8*, *IL-10*, *IL-17a*, *TNF-α*, *MCP-1 and IL-6* levels between HVs and PI-IBS (Fig. 5), and no significant difference was seen in biopsy transcript levels for a panel of pro- and anti-inflammatory cytokines (*IFN-γ*, *TNF-α*, *IL-6*, *IL-1β*, *MCP-1*, *IL-4*, *IL-10*, *IL-17f*, *IL-5*, *IL-13 and IL-2*) for PI-IBS and HVs (Fig. 6A). Furthermore, no significant differences were seen in plasma cell, B cell, CD3^+^ T cell or eosinophil numbers in biopsies from PI-IBS and HVs (Table 2, Fig. 6B).

**Table 2:** Inflammatory cell counts in HV and PI-IBS rectal biopsies.

CELL TYPE	HV (n = 9)	PI-IBS (n = 8)	Mann-Whitney p_uncorrected_	Mann-Whitney p_corrected_
B cells	43.3 ± 16.3	22.0 ± 3.3	0.32	1.92
Plasma cells	269.8 ± 41.0	265.1 ± 25.4	0.54	3.24
CD3^+^ T cells	234.0 ± 26.6	220.3 ± 48.7	0.37	0.1369
Eosinophils	35.4 ± 5.09	44.6 ± 4.1	0.17	1.02
IgE^+^ cells	91.4 ± 15.9	49.6 ± 7.1	0.036	0.216
Mast cells	134.8 ± 9.4	85.4 ± 6.7	0.001	0.006

Inflammatory cell counts in HV and PI-IBS rectal biopsies, shown as mean cell count/mm^2^ ± SEM. Mann-Whitney U test. n = 9 healthy volunteers (HV) and 8 post-infectious irritable bowel syndrome (PI-IBS) patients.

### Biopsy supernatants from PI-IBS sensitizes TRPV1 on murine DRG neurons via histamine 1 receptor

As no evidence of low grade inflammation or altered cytokine levels were detected, the potential involvement of mast cell mediators was studied. Recently we reported histamine-mediated sensitization of TRPV1 by IBS supernatants, an effect mediated by activation of histamine 1 receptor (Hrh1). To determine if a similar mechanism is present in PI-IBS, we examined the effect of incubating DRG neurons with PI-IBS supernatants, in the presence of the selective Hrh1 antagonist pyrilamine (1 µM), or in DRGs from Hrh1 knock-out mice. Pyrilamine normalized the sensitized Ca^2+^ response to capsaicin in DRGs incubated with PI-IBS biopsy supernatants (Fig. 4C), and PI-IBS supernatants were not able to potentiate the capsaicin response in DRGs collected from Hrh1 knock-out mice (Fig. 4C). These data suggest that PI-IBS supernatants contain bioactive compounds, most likely histamine or histamine metabolites that sensitize TRPV1 via Hrh1 activation.

To confirm that histamine sensitizes TRPV1, DRGs were incubated overnight with HVs supernatant supplemented with 10 µM histamine which resulted in an increased Ca^2+^ response, an effect that was prevented by pyrilamine and absent in Hrh1 knock-out mice (Fig. 4D). Moreover, as previously reported, incubation of HV submucosal neurons with histamine (10 µM) significantly increased the Ca^2+^ response to capsaicin, confirming that histamine also sensitizes TRPV1 in humans^12^. Our data indicate that the supernatant of PI-IBS sensitizes TRPV1 via a Hrh1 mediated mechanism.

Finally, we compared histamine levels and mast cell numbers in biopsy supernatants and tissue from HVs and PI-IBS patients. Surprisingly, no difference was seen in histamine concentrations (Fig. 4E), and mast cell counts were reduced in PI-IBS (Table 2; Fig. 6B, Supplementary Figure 1), suggesting that consistent with the absence of low grade inflammation, no overt increase in mast cell function occurs in PI-IBS.

## Discussion

PI-IBS patients suffer from persistent abdominal pain, lasting for many years following clearance of the initial infection. In this study, we provide direct evidence of sensitization of enteric neurons in patients 2 years after a bout of gastroenteritis. Moreover, supernatants generated from PI-IBS patient biopsies stimulate visceral colonic afferents with presumptive nociceptor function and sensitize responses to probing, indicating that an ongoing pro-nociceptive change in the gut micro-environment underlies this abdominal pain. Further investigation, demonstrates that these changes can sensitize TRPV1 channels through a Hrh1 mediated pathway. Our data for the first time provide a mechanism explanation for the abnormal pain perception in patients with PI-IBS, and suggest that histamine receptor blockade may represent a novel analgesic treatment for these patients.

PI-IBS is believed to result from a persistent low-grade inflammation of the bowel triggering symptoms of pain and altered bowel habits, by aberrant activation of intrinsic and extrinsic nerves. Enhanced cytokine signaling^4^ and increased immune cell numbers^7^ have been documented in PI-IBS^6,17–19^, however direct evidence of their role in altered neuronal signaling in patients with PI-IBS is lacking. Here, we studied PI-IBS patients from a unique cohort of residents of 2 Belgian villages, who in December 2010, were exposed to tap water contaminated with *norovirus*, *Giardia lamblia and Campylobacter jejuni* ^13^. Rectal biopsies of individuals who developed PI-IBS, all of whom continued to have complaints at the time of biopsy, 2 years after the outbreak, were studied in detail to assess the role of inflammation and neuronal sensitization in PI-IBS. Submucosal neurons from these patients displayed a significant increase in their response to the TRPV1 agonist capsaicin suggesting that long term changes in neuronal signaling occurs in PI-IBS. Submucosal neurons are involved in the regulation of secretion and blood flow in the intestine^20^, and the increased excitability of these neurons in PI-IBS is in keeping with pro-secretory changes to bowel function, consistent with the patient’s symptoms of diarrhea, and this now needs further study.

To understand the nature of the increased response to capsaicin in these neurons we examined TRPV1 transcript levels in HV and PI-IBS biopsies, observing no change. This observation suggesting that enhanced TRPV1 signaling in PI-IBS was most likely due to a sensitization of TRPV1 channel function, as opposed to an increase in channel numbers, presumably in response to an ongoing change in mediator levels within the bowel. To further explore if a pro-nocieptive change in the gut microenvironment occurs in PI-IBS we examined the effect of PI-IBS biopsy supernatants on murine afferent nerve preparations observing increased ongoing activity and sensitized responses to mechanical probing. Moreover, overnight incubation of mouse DRGs with PI-IBS supernatant resulted in an increased Ca^2+^ response to capsaicin, confirming that the PI-IBS micro-environment, even 2 years after the infection, contains mediators that activate nociceptive afferents and sensitize TRPV1. TRPV1 is involved in perception of visceral pain triggered by mechanical stimuli, as shown by impaired pain responses to colorectal distention in TRPV1 knock-out compared to WT mice^21^. Based on these findings, we propose that enhanced TRPV1 function in PI-IBS represents an important mechanism contributing to abdominal pain to bowel distention and altered bowel habits.

Sensitization of TRP channels on sensory neurons is suggested to occur via coupling with G-protein coupled receptors (GPCR) such as histamine receptors, protease receptors or cytokine receptors^22^ with subsequent activation of multiple intracellular signaling pathways including PLCβ-PI-3 and MAP kinases^23^. Hughes *et al*. elegantly showed sensitization of murine mechanosensitive colonic afferents by cytokines, particularly TNF-α^10^, however, to what extent this mechanism contributes to aberrant neuronal signaling in IBS or PI-IBS remains to be studied. In the present study, we found no evidence of increased inflammatory gene expression or infiltrating inflammatory cells in biopsies from PI-IBS patients, and no increase in biopsy supernatant cytokine levels. It should however be noted that due to the unique nature of the IBS patients studied (contaminated water supply) our sample size is inherently low, and so we should not rule out the possibility that a larger study is required to definitely confirm this observation. Nevertheless, our findings are in line with a previous study by Mearin *et al*., which also reported no difference in the number of T lymphocytes or pro-inflammatory cytokines between PI-IBS patients and infected HVs 3 years after a *Salmonella* infection, even though only PI-IBS patients were hypersensitive to rectal distention^24^. Similarly, no differences in serum cytokines and mucosal cytokine expression were detected between PI-IBS and HVs in a large Swedish cohort^25^. It is not clear why the presence of low-grade inflammation is an inconsistent finding in PI-IBS, and it is beyond the scope of this study to investigate it further. However, it is notable that the patients we have studied and those of Mearin *et al*. were examined 2–3 years after the precipitating infectious event. It may therefore be possible that low-grade inflammation is an interim event following gastroenteritis that is superseded by another more persistent change in the gut micro-environment.

Having very recently shown marked clinical efficacy of anti-histamine treatment against symptoms of pain and discomfort, and evidence for histamine mediated sensitization of TRPV1 in IBS-D^12^, we were keen to understand if a common mechanism of nociceptor activation existed in PI-IBS. Co-incubation of DRGs with the Hrh1 antagonist pyrilamine prevented TRPV1 sensitization by PI-IBS biopsy supernatants highlighting a key role for H1 receptors in TRPV1 sensitization. Moreover, histamine added to HV supernatant mimicked the effect of PI-IBS supernatant and sensitized TRPV1 responses in DRGs. In contrast, supernatant from HV biopsies had no effect on TRPV1 responses in DRGs. This histamine-mediated sensitization of TRPV1 was also blocked by pyrilamine and lacking in DRG neurons from Hrh1 knock-out mice confirming the involvement of Hrh1 activation. Despite displaying a clear histamine receptor mediated effect we could not detect a significant increase in histamine content between biopsy supernatants of PI-IBS compared to HVs, in contrast to previous studies^8,26,27^. This is likely to be a technical issue as histamine is rapidly degraded into its metabolites. Interestingly, histamine metabolites maintain the capacity to interact with H1 receptors via their aromatic ring^28^, and to sensitize TRPV1 via activation of the Hrh1^12^, explaining the ability of our PI-IBS supernatants to display stimulatory effects at Hrh1 receptors. In keeping with the overall lack of evidence for low-grade inflammation, we found decreased rather than increased mast cell numbers in biopsies of PI-IBS patients. These findings would suggest that the activity of mast cells rather than an increased number is essential in the pathophysiology of IBS and PI-IBS, as hypothesized previously^29^. Although it may be that mast cells are not the main source of histamine in biopsy samples, with the microbiome^30^, representing a significant alternative source of histamine. However, more in depth studies are required to elucidate the exact source of histamine and its metabolites.

In summary, by combining electrophysiological assessment of visceral afferents with presumptive nociceptor function, and state-of-the-art live Ca^2+^ imaging of neurons in rectal biopsies, from a well-defined group of PI-IBS patients, we have provided direct evidence for long term changes in neuronal sensitivity in patients with PI-IBS, which we believe are driven by pro-nociceptive changes to the gut micro-environment. These changes influence excitability in enteric neurons and extrinsic nociceptors, which in turn are able to drive patient symptoms of diarrhea and abdominal pain. These changes are seen in the distal gut an area remote from the initial precipitating infection (typically upper gut), and most likely represent a global pro-nociceptive change in the bowel, consistent with previous demonstrations of rectal hypersensitivity in PI-IBS patients^24^. It should be noted that this study was performed in a small cohort of PI-IBS patients, and further work in in a larger cohort of PI-IBS patients is now needed to consolidate these findings.

Significantly, although initiated by inflammation, the pro-nociceptive changes seen in PI-IBS patients are not mediated by altered immune cell numbers or inflammation. Instead, responses are mediated by Hrh1 receptor sensitization of TRPV1 signaling, suggesting that similar to “standard” IBS^12^, Hrh1 antagonism may represent an interesting new target for treatment of PI-IBS. Taken together, this is the first study using a unique cohort of PI-IBS patients showing histamine-mediated TRPV1 sensitization via Hrh1 as potential mechanism for persistent VHS observed in PI-IBS patients, in absence of low-grade inflammation. Together with the results of our previous clinical trial with the histamine 1 receptor antagonist ebastine^12^, our data provides the first evidence that peripheral histamine 1 receptor antagonism can serve as a new treatment for PI-IBS.

## Methods

### Patients

In 2010, 2 small villages in Antwerp, Hemiksem and Schelle, were exposed to contaminated tap water by which inhabitants were infected with *Giardia lamblia*, *Campylobacter jejuni* and *Norovirus* ^13,31^. Patients previously identified with PI-IBS^13^ (n = 34) were re-contacted 2 years after the outbreak. Patients who were still symptomatic and fulfilled the Rome III IBS criteria were invited to participate to this study. Of these 34 patients, 19 were asymptomatic, one did not fulfill ROMEIII criteria, 4 patients moved, one patient was pregnant and one refused to participate. Hence, eight patients (median age: 53 years, IQR: 35–54, 7 F) were included. Also 3 individuals who suffered from an infection but did not develop PI-IBS were recruited (PI-HVs) (median age: 61 years, IQR: 28–64, 0 F). Healthy volunteers (HVs, n = 9, median age: 45 years, IQR: 25–50, 4 F) were recruited by public advertisement. HVs were free of abdominal symptoms and had no history of gastrointestinal disease, no previous gastrointestinal surgery and were not on gastrointestinal medication (Table 1).

### Study design

All participants were invited to undergo a proctoscopy to collect 7 rectal biopsies (1 for immunohistochemistry, 1 for RNA analysis, 3 for Ca^2+^ imaging and 2 biopsies were incubated for 24 hours in RPMI (Lonza, Verviers, Belgium) to collect supernatants^12^). Supernatants were stored at −80 °C until use.

### Ca^2+^ imaging of human submucosal neurons

Biopsies were immediately processed to obtain the submucosal plexus, which was loaded with 1 µM Fluo-4 AM (Molecular Probes, Invitrogen, Merelbeke, Belgium) for Ca^2+^ imaging as previously described^11^. Fluorescence intensity recordings (Fi) were normalized to the starting value (Fo) and can therefore be expressed as a % of Fo. The difference (Δ) in amplitude between baseline and peak was calculated (Δ = Fmax/Fo − Fbaseline/Fo) and is therefore expressed as a %. Images were acquired at 2 Hz and collected by TILLVision software (TILL Photonics, Oberhausen, Germany). Data were analyzed by custom-written macros in IGOR-PRO (Wavemetrics, Lake Oswego, Oregon, USA)^12^. The response of submucosal neurons to capsaicin perfusion (1 ml/min for 5 seconds, 0.1-1-10 nM) (Sigma-Aldrich®, Diegem, Belgium) was compared between HVs, PI-HVs and PI-IBS patients. One µM TRPV1 antagonist SB-366791 (Tocris Bioscience, Bristol, United Kingdom) was used to block the capsaicin response.

### Ca^2+^ imaging of murine dorsal root ganglia neurons

Lumbosacral (L5-S2) DRG neurons from 3 to 4 adult (8–12 weeks) wild type or Hrh1 knock-out mice were bilaterally excised under a dissection microscope, digested and cultured in complete medium (260 µl) on poly-D-lysine/laminin-coated glass coverslips as previously described^32^. Supernatants (140 µl) derived from either HVs or PI-IBS patients, combined or not with 10 µM histamine (Sigma-Aldrich®, Diegem, Belgium) and/or 1 µM Hrh1 antagonist pyrilamine (Sigma-Aldrich®, Diegem, Belgium), was added overnight. Cultured DRG neurons were subsequently loaded with 2 µM Fura-2AM (Invitrogen, Ghent, Belgium) for 20 minutes at 37 °C.

Intracellular Ca^2+^ concentration ([Ca^2+^]_i_) was measured and quantified as previously described^12,32^. Neurons were identified by a Krebs–based solution in which the KCl concentration was increased to 45 mM by iso-osmotic substitution of NaCl. The baseline was monitored for 120 s and the chamber was thereafter perfused with 10 nM capsaicin (Sigma-Aldrich®, Diegem, Belgium). TRPV1 positive DRG neurons were identified by a high dose of capsaicin (1 µM).

### Afferent nerve recording

These experiments were performed at Queen Mary University of London. Experiments were performed using colons from 12 weeks old male C57BL/6 mice (Charles River UK, Ltd.). Single unit afferent nerve activity was recorded from teased lumbar splanchnic nerve fibers innervating the distal colon, as previously described^33^. Receptive fields were identified and characterized based on the criteria previously developed by Brierley *et al*.^34,35^. Only colonic afferents with presumptive nociceptor function were used. Mechanosensitivity was determined by repeated probing of the receptive field with a 0.6 g von Frey hair (vFh). Thereafter, rectal biopsy supernatants (100 µl) were applied for 12 minutes and mechanosensitivity was re-assessed (0.6 g vFh) post-supernatant. For data analysis, individual single unit discharge was discriminated using template matching wavemark software. For mechanosensitivity to vFh probing, the peak action potential discharge (Hz) over a 2 s period within the 3 s period of probing was determined for each probe and a mean response calculated from each set of 3. For chemosensitivity to biopsy supernatants, nerve discharge of individual fibers was expressed as a rate histogram (20 s) bin width. The peak change in activity during supernatant application was normalized to baseline (mean firing rate over 200 s prior to incubation).

### RNA extraction and RT-qPCR

RNA was extracted from 5–10 mg colonic biopsies using RNeasy minikit (Qiagen, Hilden, Germany). cDNA was prepared from 2 µg total RNA using qScript cDNA supermix (Quanta Biosciences, Gaithersburg, Maryland, USA) according to the manufacturer’s instructions. RT-qPCR was performed to quantify inflammatory (IL-1β, IFN-ɣ, TNF-α, IL-2, IL-4, IL-5, IL-6, IL-10, IL-13, IL-17f, MCP-1) and neuronal TRPV1 marker mRNA expression using Probe Master Mix (Sigma-Aldrich®, Diegem, Belgium) or FastStart Essential DNA Green Master (Roche GmBH, Mannheim, Germany) (primer and probe sequences are listed in Supplementary Table 1). Gene expression was normalized to an endogenous reference gene, β-actin and relative gene expression was calculated as 2^−∆∆Ct^ ^36^.

### Immunostaining of rectal biopsies

Immune infiltration was assessed in rectal biopsies using standard immunohistochemistry. Biopsies were fixed in 4% paraformaldehyde (Sigma-Aldrich®, Diegem, Belgium) and put through a sucrose gradient before embedding in OCT-compound (Sakura Finetek, Antwerp, Belgium). Eight µm cryosections were incubated with 1% bovine serum albumin (BSA) (SERVA Electrophoresis GmbH, Heidelberg, Germany) in phosphate-buffered saline containing 0.3% Triton-X (Thermo Fisher Scientific Inc., Leusden, The Netherlands) for 60 min at room temperature (RT) to prevent non-specific antibody binding, followed by overnight incubation with the primary antibody at 4 °C. The following pimary antibodies were used for B cells (1:250 anti-CD20cy L26 (Dako Belgium nv/sa, Heverlee, Belgium)), CD3^+^-T cells (1:100 anti-CD3 [CA-3] ab82251 (Abcam, Cambridge, United Kingdom)), eosinophils (1:500 anti-major basic protein BMK13 (Merck Millipore, Solna, Sweden)), plasma cells (1:30 anti-Syndecan-1 [B-A38] (Abcam, Cambridge, United Kingdom)), immunoglobulin E^+^ (IgE^+^) (1:250 mouse anti-human MHE-18 (BioLegend, Fell, Germany)) and mast cells (1:500 anti-CD117 K963 (Sanbio B.V., Uden, The Netherlands). Plasma cells were detected after heat-mediated antigen-retrieval (2 × 5 minutes at 500 watt) in citrate buffer (pH = 6). For eosinophil staining, sections were pre-treated with 0.1% hydrogen peroxide (VWR, Leuven, Belgium) (10 minutes, RT). After wash, sections were incubated with secondary antibodies (1 hour in the dark, RT) and mounted in SlowFade® Gold (Life Technologies Europe B.V., Gent, Belgium).

### Cytokine analysis in supernatants

Biopsy supernatant was collected and stored at −80 °C until analysis as described above. Cytokine levels of IL-4, IL-13, IFN-g, IL-1b, CXCL8, IL-10, IL-17a, TNFα, MCP-1 and IL-6 were quantified by cytometric bead array (BD Bioscience, Erembodegem, Belgium). Samples were acquired on FACSCanto flow cytometer (BD Bioscience) and analysed by FCAP v3.0 analysis software (Soft Flow Inc., Pecs, Hungary).

### Enzyme linked immunosorbent assay (ELISA)

Histamine and tryptase levels in rectal biopsy supernatants were assessed by a commercially available histamine (IBL International GmbH, Hamburg, Germany) and tryptase (MyBioSource, San Diego, USA) kit, respectively, according to the manufacturer’s instructions.

### Statistics

All statistical analyses were performed using Graphpad Prism (La Jolla, USA). Continuous data were summarized by their mean and standard deviation. When deviations from normality were observed, medians and interquartile values were presented. Comparisons between groups were made using a t-test or Wilcoxon rank-sum test, as appropriate. Statistical significance is assumed when p ≤ 0.05 after Bonferroni correction for multiple testing.

Statistical analyses of the peak F340/380 ration for the Ca^2+^ imaging experiments were performed after correction for the individual baseline Ca^2+^. All values are expressed as means ± SEM from n mice or n cells treated with each of the individual supernatants. Statistical comparisons between 2 groups were performed by a Wilcoxon signed rank test or Mann–Whitney U test as appropriate or ANOVA when comparing more than 2 groups. Categorical data were analyzed by the Fischer’s exact test.

### Study approval

All animal experiments were carried out in accordance to the European Community Council guidelines and were approved by the local ethics committee of the KU Leuven (ECD P157/2014) or the UK Animals Scientific Procedures Act (1986). The human study was approved by the Medical Ethical Committee of the University Hospital of Leuven (study protocol S51573) and all experiments were performed in accordance with relevant guidelines and regulations. All participants gave written informed consent.

### Data availability

The datasets generated during and/or analyzed during the current study are available from the corresponding author on reasonable request.

## Electronic supplementary material

Supplementary information
