# Supplementary material for: Evidence for long-term sensitization of the bowel in patients with post-infectious-IBS
Source: Sci Rep. 2017 Oct 19;7:13606. doi: 10.1038/s41598-017-12618-7 (PMC5648751; doi:10.1038/s41598-017-12618-7)
Supplement: Supplementary file 1 — Supplementary information [file 41598_2017_12618_MOESM1_ESM.pdf]

# Supplementary Information:

# Evidence for long-term sensitization of the bowel in patients with post-infectious-IBS

Balemans D[1&], Mondelaers SU[1&], Cibert-Goton V[2&], Stakenborg N[1], Aguilera-Lizarraga J[1], Dooley J[3], Liston A[3], Bulmer DC[2,4], Vanden Berghe P[1], Boeckxstaens GE[1§]*, Wouters MM[1§]

&; §: authors contributed equally

1 Translational Research Center for Gastrointestinal Disorders, Dept. of Clinical and Experimental Medicine, KU Leuven, Leuven, Belgium

2 National Centre for Bowel Research and Surgical Innovation, Centre for Neuroscience and Trauma, Blizard Institute, Bart's and the London school of Medicine and Dentistry, Queen Mary University of London, London, UK

3 Autoimmune Genetics Laboratory, VIB and Department of Microbiology and Immunology, KU Leuven, Belgium

4 Department of Pharmacology, University of Cambridge, Cambridge, UK

<u>**Supplementary figures and legends**</u>

**Supplementary Figure 1**

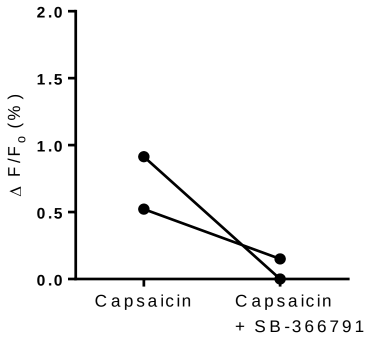


<u>**Supplementary Figure 1:**</u> **Capsaicin-induced response in IBS patients is blocked by TRPV1 antagonist SB-366791.** Data showing the amplitude of the $Ca^{2+}$ flux (%) to 0.1 nM capsaicin in IBS patients (n=2) before and after incubation with TRPV1 antagonist SB-366791.

**Supplementary Figure 2**

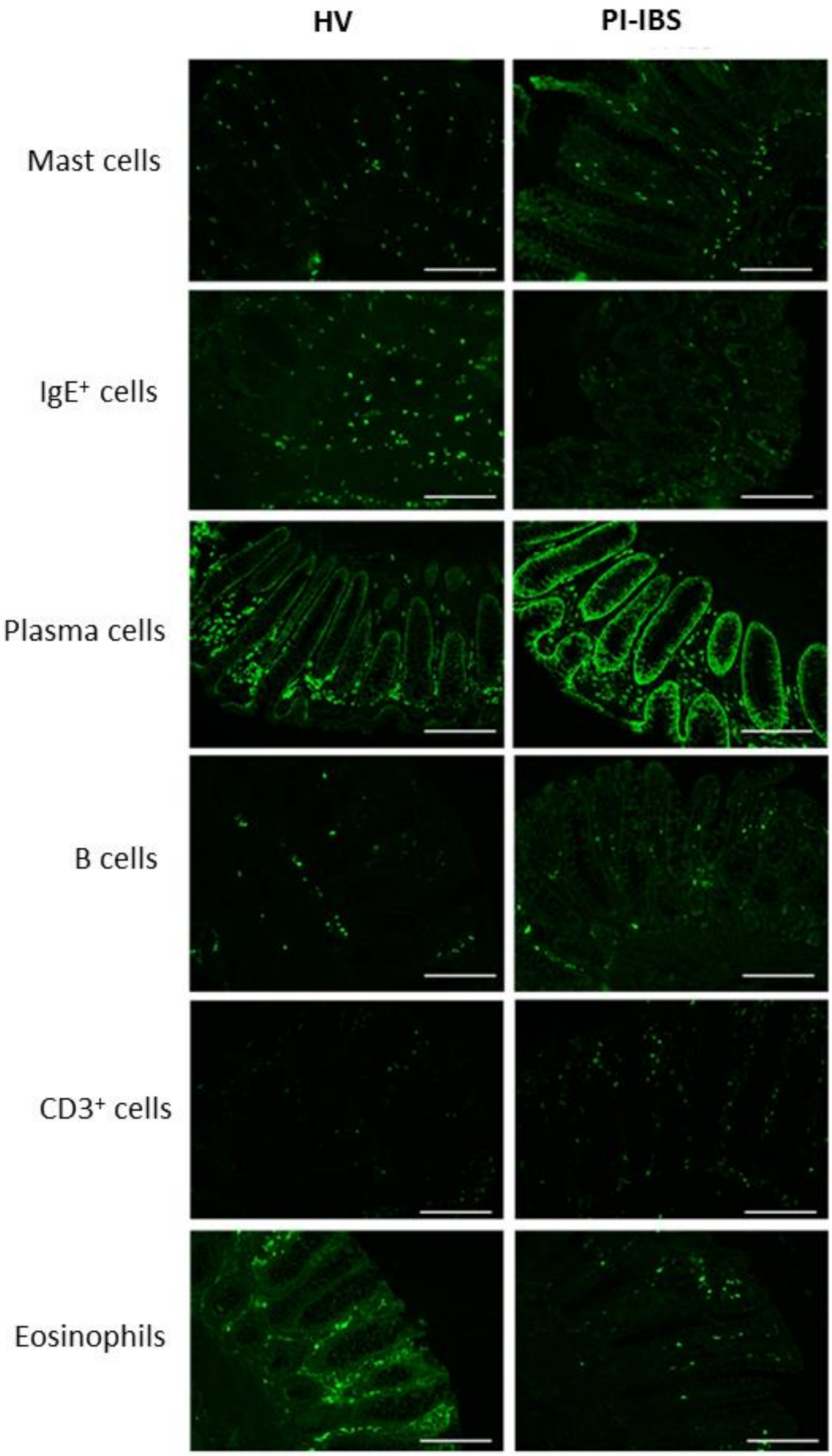


<u>Supplementary Figure 2</u>: **No evidence for low-grade inflammation in rectal biopsies of PI-IBS patients.** Immunohistochemical staining of mast cells, IgE$^{+}$ cells, plasma cells, B cells, CD3$^{+}$ T cells and

eosinophils in HV (n=9) and PI-IBS (n=8) rectal biopsies (as indicated). HV = healthy volunteer, PI-IBS = post-infectious IBS. Scalebar=200 µm.

## Supplementary Tables

**Supplementary Table 1: Primer sequences for gene detection by RT-qPCR.**

| Gene | Protein | Forward (5’ -> 3’) | Reverse (3’ -> 5’) | Probe |
|---|---|---|---|---|
| Actb | β-actin | ACTCCATCATGAAGTGTGA | GGCAGTGATCTCCTTCTG | TGTACGCCAACACAGTGCTG |
| IL1 beta | IL-1β | AGCTACGAATCTCCGACCAC | CGTTATCCCATGTGTCGAAGAA | - |
| IL2 | IL-2 | TAACCTCAACTCCTGCCACAA | GAGCATCCTGGTGAGTTTGG | - |
| IL4 | IL-4 | ACTGCACAGCAGTTCCACAG | ACTCTGGTTGGCTTCCTTCAC | - |
| IL5 | IL-5 | TCTACTCATCGAACTCTGCTGA | CCCTTGCACAGTTTGACTCTC | - |
| IL6 | IL-6 | TCTGGATTCAATGAGGAG | CTGGATCAGGACTTTTGTA | CTGGCTTGTTCCTCACTACTCTCA |
| IL10 | IL-10 | AAGCCATGAGTGAGTTTG | GTCCTAGAGTCTATAGAGTCG | CGTATCTTCATTGTCATGTAGGCTTCT |
| IL13 | IL-13 | AGCCCTCAGGGAGCTCAT | CTCCATACCATGCTGCCATT | - |
| IL17f | IL-17f | CCTACACTGGGCCTGTACAAC | ATGAAGCTTGACATTGGCATC | - |
| IFNg | IFN-γ | ACGAGATGACTTCGAAAA | TTCGCTTCCCTGTTTTAG | CGGTAACTGACTTGAATGTCCAACG |
| TNF | TNF-α | GACGAACATCCAACCTTC | GGTGGTCTTGTTGCTTAA | CCCCTGCCCCAATCCCTTTA |
| *Ccl2* | MCP-1 | CCCCAGTCACCTGCTGTTAT | TCCTGAACCCACTTCTGCTT | ATTGTGGCCAAGGAGATCTG |
| *TRPV1* | TRPV1 | CCCCGATAGCTCCTACAACA | GCAGCAGGATGATGAAGACA | - |

sTable 1 summarizing the gene, the protein and corresponding primer sequences.
